# Evidence for Separate Contributions of High and Low Spatial Frequencies during Visual Word Recognition

**DOI:** 10.3389/fnhum.2017.00324

**Published:** 2017-06-22

**Authors:** Kurt Winsler, Phillip J. Holcomb, Katherine J. Midgley, Jonathan Grainger

**Affiliations:** ^1^NeuroCognition Laboratory, Department of Psychology, San Diego State UniversitySan Diego, CA, United States; ^2^Laboratoire de Psychologie Cognitive, CNRS and Aix-Marseille UniversitéMarseille, France

**Keywords:** spatial frequency, word recognition, masked priming, ERP, N400, N250

## Abstract

Previous studies have shown that different spatial frequency information processing streams interact during the recognition of visual stimuli. However, it is a matter of debate as to the contributions of high and low spatial frequency (HSF and LSF) information for visual word recognition. This study examined the role of different spatial frequencies in visual word recognition using event-related potential (ERP) masked priming. EEG was recorded from 32 scalp sites in 30 English-speaking adults in a go/no-go semantic categorization task. Stimuli were white characters on a neutral gray background. Targets were uppercase five letter words preceded by a forward-mask (#######) and a 50 ms lowercase prime. Primes were either the same word (repeated) or a different word (un-repeated) than the subsequent target and either contained only high, only low, or full spatial frequency information. Additionally within each condition, half of the prime-target pairs were high lexical frequency, and half were low. In the full spatial frequency condition, typical ERP masked priming effects were found with an attenuated N250 (sub-lexical) and N400 (lexical-semantic) for repeated compared to un-repeated primes. For HSF primes there was a weaker N250 effect which interacted with lexical frequency, a significant reversal of the effect around 300 ms, and an N400-like effect for only high lexical frequency word pairs. LSF primes did not produce any of the classic ERP repetition priming effects, however they did elicit a distinct early effect around 200 ms in the opposite direction of typical repetition effects. HSF information accounted for many of the masked repetition priming ERP effects and therefore suggests that HSFs are more crucial for word recognition. However, LSFs did produce their own pattern of priming effects indicating that larger scale information may still play a role in word recognition.

## Introduction

It is certainly not an overstatement to say that human perceivers are generally unaware of the speed and complexity of the neuro-cognitive mechanisms underlying visual recognition. Perhaps nowhere is this truer than in the case of visually encountered words where skilled readers regularly recognize and understand as many as 300 items per minute. While in recent decades substantial gains have been made in our understanding of the nature of the neuro-cognitive networks involved in reading, there is still a lot that is not known. One thing that is clear is that reading in general and word recognition in particular is a comparatively new skill (for example, compared to face recognition). And as such, it has not had enough time evolutionarily speaking to have resulted in specialization of early visual processing centers specifically for recognizing words (Dehaene and Cohen, 2011). The most likely explanation for how readers become experts in visual word processing is that early intense exposure to print during a crucial period of development allows brain areas in temporo-occipital regions, which might otherwise be used for some other visual expertise, to be tuned to efficiently process letters and letter combinations (the neural recycling hypothesis, Dehaene, 2009). However, how these areas become tuned for the printed word and the exact nature of the processing executed in this region of the brain is still unknown.

Similar to how acoustic signals can be represented by a series of auditory frequencies, visual information, including written words, can be represented with spatial frequencies—repeating cycles of luminance information. It is now well accepted that the human visual system codes visual information using spatial frequency information (De Valois and De Valois, 1990). Starting at retinal ganglion cells which project to the magnocellular and parvocellular layers in the lateral geniculate nucleus and onward to various other visual areas, two visual processing pathways are formed which ultimately interact to perceive visual information. The magnocellular pathway, sometimes associated with the “where” or dorsal stream, quickly conveys coarse-grained low spatial frequency (LSF) information about the location and global shape of a stimuli. Conversely the parvocellular pathway, which is more associated with the “what” or ventral stream, takes longer, but communicates more precise fine-grained high spatial frequency (HSF) information. In a behavioral sense, the presence of both pathways has clear evolutionary payoffs; high quality imagery costs processing time, yet there are instances when precise information about an object or a scene is irrelevant, and quick rough visual feedback is more advantageous to perform the correct behavior (e.g., having to react to a threat).

When it comes to more specialized functions of the visual system, it seems these dual pathways help enable the incredible efficiency of recognition processes. While it may be evident that HSF information is necessary for making precise judgments about a stimulus, the role of LSFs has been a topic of ongoing research. It has been suggested that during visual perception as a first pass, the magnocellular pathway rapidly projects coarse information to higher level visual areas, which in turn feedback information into lower level areas to help with processing (Bullier, 2001). Following from this, Bar’s influential model of object recognition proposes that these LSF magnocellular projections are used to formulate rough guesses about possible identities of an object which is then relayed top-down to limit the number of possible candidates that need to be considered by more precise bottom up processes (Bar, 2003). This model has been supported with functional imaging results implicating the orbitofrontal cortex with LSF processing which feeds top-down information to the fusiform gyrus at the end of parvocellular ventral stream (Bar et al., 2006; Kveraga et al., 2007). The importance of LSF information has also been demonstrated for the analysis of scenes where LSF information is critical for rapid categorization (Schyns and Oliva, 1994) and is processed before HSF information (Peyrin et al., 2010), however there is also evidence that these systems are flexible and HSFs may also be available at early stages depending on the task demands (Oliva and Schyns, 1997). Further, holistic face processing has been shown to be reliant on LSFs (Goffaux and Rossion, 2006) and LSFs are processed before HSFs during face recognition (Goffaux et al., 2011). Also interestingly, Vuilleumier et al. (2003) found that HSF and LSF information project to different brain areas when processing fearful facial emotions; HSFs activating the fusiform cortex, associated with the actual identity of the face, and LSFs activating the amygdala and other areas associated with the emotional content of the face for faces displaying fear. Overall it seems that LSFs are important for making fast global inferences, which may in turn increase the efficiency of more precise analysis.

But what about the recognition of other complex visual stimuli such as written words? Efficiency is certainly essential, but most written words occupy much less of the observer’s visual angle than objects, faces, or scenes and distinctions between letters and words can be very subtle. Perhaps LSF information about word-shape is not useful, and only fine-grained HSF analysis of smaller features like letters is important during word recognition. Indeed, there is significant evidence that letter identification, instead of word shape, is more critical to the recognition of words (e.g., Paap et al., 1984; Perea and Rosa, 2002; Pelli et al., 2003; but see Beech and Mayall, 2005). Most recent models of visual word recognition rely on primarily feature or letter based approaches to word identification (e.g., Whitney, 2001; Grainger and Van Heuven, 2003; Davis, 2010) rather than word shape information. One argument for the preeminence of letter-based processing is that given the problem of shape invariance (e.g., different fonts), it makes more sense for the brain to learn a set of 26 letters in all their forms rather than the shapes of tens of thousands of words in all their forms (Grainger, 2008; Grainger and Dufau, 2012). For single letters, Fiset et al. (2008) showed that identification is most reliant on line termination rather than larger shape features, suggesting that even within letters, smaller HSF-carried features such as line terminations are more important than larger features. Another line of research using behavioral, neuroimaging and neuropsychological methods has shown that, the left hemisphere has a bias for HSF information (e.g., Woodhead et al., 2011; Roberts et al., 2013; Tadros et al., 2013; Fintzi and Mahon, 2014). Additionally, the overall left hemisphere bias for HSFs was not found to exist in children who have not yet learned to read indicating that the bias emerges concurrently with, or perhaps as a function of learning to read (Ossowski and Behrmann, 2015).

While there is strong support that letters and thus HSF channels are the most relevant when it comes to word processing, there is nevertheless evidence that word shape and perhaps by extension LSF information is being used by the word recognition system. Healy and Cunningham (1992) found that during proof reading, misspellings that affected word shape were more likely to be caught than misspellings that did not change word shape, in both experienced and learning readers. Also, Allen et al. (1995) showed a larger disadvantage for the identification of words compared to non-words when word shape was manipulated by presenting words in mixed-case. Further, Lété and Pynte (2003) found that among less frequent words, words were responded to faster if they had ascenders or descenders (thus a more distinct word-shape) compared to neutral words. There is also evidence that the most useful visual features for word recognition are towards the edges of words rather than the middle, again suggesting that larger scale word shape information might play an important role in visual word recognition (Beech and Mayall, 2005). Recently, Jordan et al. (2016) found evidence that during sentence reading, skilled readers made better use of particularly LSFs compared to less skilled readers, further indicating that LSFs may be important for efficient reading.

Another major line of research linking LSF information and word recognition is from studies of dyslexia. It has been found that individuals with dyslexia tend to have reduced sensitivity to middle-to-LSFs (e.g., Lovegrove et al., 1980; Mason et al., 1993; but see Skottun, 2000). Even for non-dyslexic individuals, it has been found that lower-skilled compared to higher-skilled readers show a similar reduction in sensitivity to-LSFs (Patching and Jordan, 2005). Additionally, post mortem studies of dyslexic brains have revealed that magnocellular layers in their LGNs were on average 30% smaller than in the control brains (Livingstone et al., 1991; Galaburda and Livingstone, 1993) and imaging studies have found disruption in dyslexic brains for motion processing in area MT/V5 (Eden et al., 1996; Eden and Zeffiro, 1998), an area thought to be fed primarily by magnocellular inputs. This evidence has led to theories implicating a deficit in magnocellular or LSF information processing as a factor contributing to at least one subtype of dyslexia (Lovegrove, 1993; Stein and Walsh, 1997). The research on dyslexia and magnocellular pathways add support to the possibility that LSFs are contributing in some way to the process of word recognition, although the connection between the two may be mediated by other functions of magnocellular networks such as visual orienting during reading, rather than global word shape analysis (for more on the magnocellular theory of dyslexia, see Stein, 2001).

The purpose of the current study is to further investigate the roles of both HSFs and LSFs during the process of word recognition using the temporal precision of event-related potentials (ERPs) combined with the masked repetition priming paradigm. Masked priming involves the brief presentation of a prime word which is concealed by some combination of forward and backwards masking stimuli, which precede and follow the prime respectively. A visible target word immediately follows the masked prime. Because the prime is very brief and is “masked” it is not consciously perceived although an abundance of evidence suggests that it is nevertheless processed to some extent as reflected in changes in the processing of the target stimulus. Depending on the relationship (e.g., orthographic, lexical or semantic) between the prime and target, various effects can be observed in both behavioral and ERP measures on the target word. This paradigm is the gold standard approach in the word processing literature for untangling the cascade of processes involved in recognizing visually presented words (e.g., Forster et al., 2003).

The most straightforward type of masked priming is repetition priming in which a target word is primed by the same repeated word (e.g., table-TABLE), or an unrelated word (e.g., truck-TABLE). Behaviorally, participants make faster judgments about the target word if it was primed with a repetition (e.g., Forster and Davis, 1984), or even with a word that shares letters with the target word (e.g., tr%ck-TRUCK, Grainger and Jacobs, 1993). With ERPs, repetition was initially found to attenuate (indicating less processing for) the well-known N400 component (Schnyer et al., 1997; Misra and Holcomb, 2003) which is thought to index lexical semantic processing (e.g., Holcomb, 1993) or more specifically, the processes of mapping lexical forms onto semantic representations (Grainger and Holcomb, 2009). More recently, by decreasing the long prime-target stimulus onset asynchronies of prior studies from 500 ms to 50–70 ms, Holcomb and Grainger (2006) revealed a number of earlier ERP effects to masked repetition priming in addition to the N400, and these components have been mapped on to various stages of word recognition in the bi-modal interactive activation model (Grainger and Holcomb, 2009). The earliest of these components is the N/P150, a bifocal effect that is more positive to repeated words at occipital sites and more negative in frontal sites. This effect probably represents a location specific feature-to-letter mapping process (Grainger and Holcomb, 2009), as it is only sensitive to featural overlap (Petit et al., 2006; Chauncey et al., 2008), and is not affected if repeated primes are shifted so that their letters do not overlap with the corresponding letters of the target word (Dufau et al., 2008). Subsequent to the N/P150, masked repetition priming also elicits an N250 effect, a greater negativity to unrelated compared to repeated words which is distributed across the midline of the scalp but tends to be the largest over anterior sites (Holcomb and Grainger, 2006; Kiyonaga et al., 2007). Unlike the N/P150, the N250 is not dependent on exact feature overlap (Dufau et al., 2008) and can also be modulated by related non-words such as transposed letter primes and pseudohomophones (e.g., barin-BRAIN and brane-BRAIN respectively—Grainger et al., 2006). Thus, the N250 effects may represent influences of mapping location invariant features like letters or bigrams onto word representations as proposed in the bi-modal interactive activation model (Grainger and Holcomb, 2009). Following the N250, the typical N400 effect is usually seen with larger negativities to targets unrelated to their primes compared to targets that are repetitions. Such N400 effects are usually interpreted as reflecting the mapping of whole-word representations onto meaning representations (Grainger and Holcomb, 2009).

**Figure 1:**
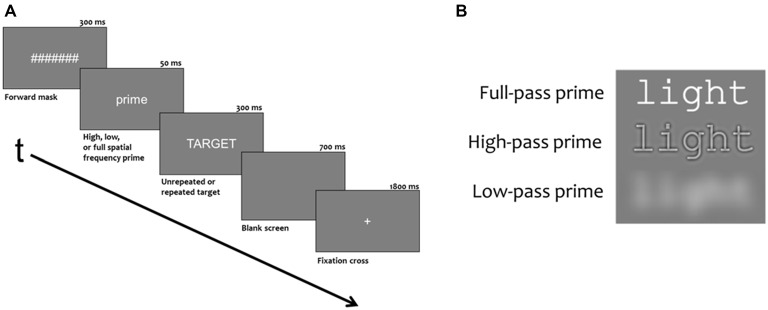
**(A)** Demonstration the duration and order of stimuli presented during each trial. **(B)** Example of a prime with different spatial frequencies filtered out.

Here, we used a similar masked repetition priming paradigm as Grainger et al. (2012), but in addition we also manipulated the spatial frequency content of the masked prime words. Masked primes contained either the full range of spatial frequencies (FSF primes), only high spatial frequencies (HSF primes, >15.2 cycles/deg) or only low spatial frequencies (LSF primes, <3.7 cycles/deg). Target words, and the masking stimuli (a row of hash marks) were always presented as FSF stimuli (see Figure 1). The logic of the spatial frequency manipulation is that if either HSF or LSF primes are able to produce some or all of the typical ERP masked priming effects seen with FSF primes, this could be interpreted as strong evidence that these frequencies are used by the word recognition system. Like a number of other masked repetition priming studies (e.g., Morris et al., 2010; Grainger et al., 2012; Massol et al., 2012; Experiment 2), the backwards mask for the prime was a case change between the prime and the target. This is done to avoid having to separate the prime-target pair with an additional stimulus or added inter-stimulus interval (ISI). Also, the case change emphasizes the prime’s effect at levels beyond the physical similarity between the prime and target. Though some studies have shown form-level N/P150 effects using a case change between prime and target (e.g., Holcomb and Grainger, 2006), this effect seems to rely on a large degree of featural overlap between prime-target pairs. While this case change reduces the chance of purely feature-based priming effects like the N/P150, the current study is more focused on issues at the sub-lexical (letter) and lexical levels of processing. Specifically, this experiment was designed to test the ability for different spatial frequency primes to activate the lexical representation of the target word. Evidence of this may be found as priming effects on the N250 and N400.

As in Grainger et al. (2012), we also manipulated the lexical frequency of prime and target words such that half of all pairs were of high or low lexical frequency. Lexical frequency refers to how often a word is used in a language, and has been shown to have a potent influence on word processing. For instance, lower frequency words take longer to recognize and generate larger N400s (e.g., Van Petten and Kutas, 1990). This pattern is sometimes thought of as reflecting higher resting state activation, or lower activation thresholds, for high lexical frequency words (Marslen-Wilson, 1990). Examining the influence of lexical frequency on priming as a function of spatial frequency is another way to determine whether there are differential effects of LSFs and HSFs during word recognition. If lexical frequency interacts with the priming capabilities of a certain spatial frequency, this may indicate that this spatial frequency affected processing in the typical lexico-semantic framework that gives rise to lexical frequency effects. Conversely, no interaction with the priming effects might indicate priming in a different network, potentially more related to form. In keeping with previous research, we predicted an attenuation of the N250 and N400 components time-locked to target words preceded by the same (repeated) as compared to a different (unrelated) prime word when the prime word contained FSF information. Given the evidence that visual word recognition brain areas are more sensitive to HSF information, we also predicted N250 and N400 repetition priming for HSF primes but comparatively weaker N250/N400 priming effects for LSF primes. Moreover, for the less salient HSF and LSF primes, priming effects may be more clearly seen for high lexical frequency prime-target pairs than low lexical frequency pairs due to their greater familiarity. Additionally, because of the faster processing of LSF information it is possible that LSF primes would produce earlier evidence of masked priming than HSF primes.

## Materials and Methods

### Participants

Research was carried out in accordance with the San Diego State University Institutional Review Board and all subjects gave written, informed consent in accordance with the Declaration of Helsinki. The protocol was approved by the San Diego State University Institutional Review Board. A total of 34 participants volunteered for this study, however four were eliminated from the final analysis due to too many trials exceeding artifact rejection criteria (>20% of total trials). The 30 remaining participants ranged in age from 18 to 28 years (mean age = 22.8 years old [SD = 2.73]) and included 20 females. Most were students at San Diego State University, compensated with $15 dollars per hour for their participation. All participants reported being right handed, native English speakers with normal or corrected to normal vision with no neurological impairment.

### Stimuli

Experimental blocks had 276 trials, consisting of 240 critical trials and 36 probe trials (animal names). Of the 36 probe trials, 24 trials had the animal probe in the target position and 12 trials had the animal probe in the prime position. Stimuli were white characters on a neutral gray background presented in the Courier fixed width font (6 cm × 1 cm). Stimuli were viewed from 150 cm so primes and targets subtended 2.3 degrees of horizontal and less than 1 degree of vertical visual angle. As shown in Figure 1, trials began with a 1800 ms fixation cross (+) in the center of the screen which was followed by a rapid succession of three stimuli: a 300 ms forward mask of seven hash marks (#######), a prime word for 50 ms and a target word for 300 ms. A final 700 ms blank screen ended each trial. Targets were five letter uppercase words and primes were five letter lowercase words that were either the same word as the target (repeated) or a word unrelated to the target (unrepeated). In both conditions, one third of the primes were spatially filtered such that they contained only high frequencies, (>15.2 cycles/deg or >35 cycles/image) only low frequencies (3.7 cycles/deg or <8.5 cycles/image) or full spatial frequencies (for examples, see Figure 1).

There were three experimental blocks which were counterbalanced such that every target would be in both the repeated and unrelated condition and in each spatial frequency condition over three participants (each participant saw one experimental block). Unrelated prime-target pairs did not overlap any letters, have any obvious semantic relationship, or have any clear phonological relationship (e.g., rhyme). Also, within each condition, half of the prime-targets pairs were high frequency words (mean log HAL frequency = 11.06, range 9.3–13.7) and half were low frequency words (mean log HAL frequency = 5.83, range 4.14–7.03). See Burgess and Lund (1997) for a discussion of the HAL frequency measure. For the words in the current study, this measure was obtained from the English Lexicon Project (Balota et al., 2007).

### Procedure

Participants were seated in a comfortable chair, 150 cm from a stimulus monitor with a refresh rate set at 100 Hz in a sound attenuating darkened room. The testing session began with a short practice block, followed by one of the three counterbalanced experimental blocks. Participants performed a go/no go semantic categorization task in which they were instructed to push a button on a game controller whenever the name of an animal was presented. These probe stimuli made up approximately 13% of trials. On average every 15 trials, an icon was displayed on the screen to indicate extra time for the participant to move or blink. There were also three longer rest breaks where the participant would push a button when ready to continue the experiment.

### EEG Recording

**Figure 2:**
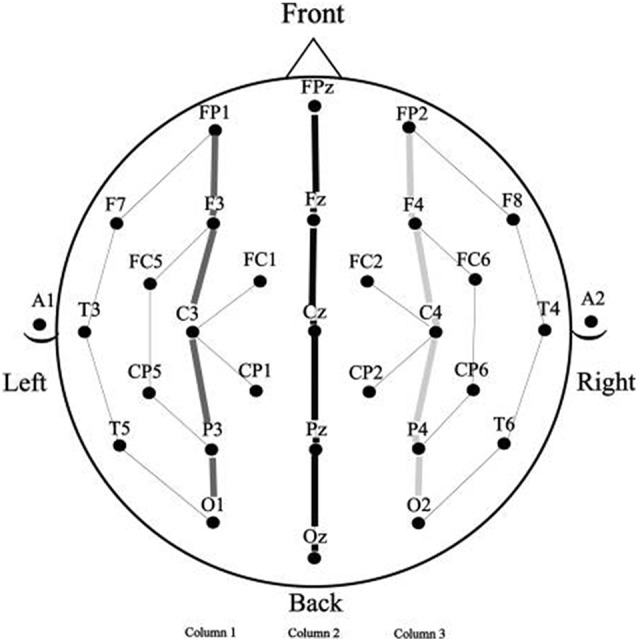
Electrode montage (standard 10–20 system) with the columns used for analysis.

Electroencephalograms were collected using a 29-channel, tin electrode cap (Electro-Cap International, Inc., Eaton, OH, USA), arranged in the International 10–20 system (see Figure 2). Electrodes were placed next to the right eye to monitor horizontal eye movements and below the left eye to monitor vertical eye movements and blinks. An electrode was placed behind each ear on the mastoid bone, the left mastoid site was used as an online reference for the other electrodes and the right mastoid site was used to evaluate differential mastoid activity. Impedance was kept below 2.5 kΩ for all scalp and mastoid electrode sites and below 5 kΩ for the two eye channels. The EEG signal was amplified by SynAmpsRT amplifier (Neuroscan-Compumedics, Charlotte, NC, USA) with a bandpass of DC to 200 Hz and was continuously sampled at 500 Hz. The stimuli and behavioral responses were simultaneously monitored by the data collection computer.

### Data Analysis

Averaged ERPs time-locked to target word onset were created off-line, using the left mastoid electrode as a reference. Inspection of the active right mastoid lead did not indicate any asymmetrical mastoid activity for any of the variables so the data were not re-referenced. Trials with muscular or ocular artifact were rejected prior to averaging.

Because the prime stimuli were physically quite different in the three spatial frequency conditions it seemed unwise to directly compare target ERPs as a function of spatial frequency. This is because ERPs are highly sensitive to the kinds of physical differences that result from manipulations like spatial frequency (for a discussion of the effects of differences in physical attributes on ERP components, see Luck, 2014). The problem of comparison across spatial frequencies arises because the prime and target stimuli in masked priming occur in the same temporal epoch (only 50 ms separate prime and target onset), so examination of target ERPs is confounded by the presence of ERP components from the prime which are temporally overlapping. For this reason, comparisons in masked priming using ERPs require that all prime and target stimuli have the same physical attributes which is the case for the repeated and unrelated items within, but not between spatial frequency conditions. To avoid this problem we therefore analyzed each spatial frequency condition was separately. Mean amplitude measurements (baselined between −100 ms and target onset) were taken in five time windows representative of components modulated by masked repetition priming. The first was a 125–175 ms epoch, widely used to measure the N/P150 (e.g., Holcomb and Grainger, 2006, 2007; Morris et al., 2008). Similar to a number of other masked priming studies (e.g., Grainger et al., 2006; Morris et al., 2008, 2010), the N250 was measured in two epochs, an early epoch from 175 ms to 225 ms which has proven sensitive to the early phase of this component, and a later 225–275 ms epoch that has shown sensitivity to the peak and trailing edge of this component. For similar reasons N400 measurements were also taken in two epochs, an early 300–400 ms epoch and a later 400–500 ms epoch. A further rationale for breaking both the N250 and N400 into separate sub-epochs is the likelihood that the spatial frequency manipulation might alter the time-course of the component processes involved in word recognition. Given the evidence that the parvocellular and magnocellular pathways may have differing temporal properties (e.g., Kauffmann et al., 2014), if the current spatial frequency manipulation affects processing in these separate pathways then the time frames of repetition effects may be altered. Single large time measurements for N250 and N400 epochs may not be sensitive to subtle timing differences and thus smaller windows were used to better capture possible differences in timing. Repeated measure Analysis of Variance (ANOVAs) were performed on these measurements for each spatial frequency condition (Full, High and Low). Factors included Repetition (Repeated or Unrepeated), Lexical Frequency (High or Low) and two distributional factors which used 15 representative electrode sites arranged in a 3 × 5 grid (see columns indicated in Figure 2); Laterality (Left, Midline, Right) and Anteriority (occipital, parietal, central, centro-frontal and frontal).

As mentioned above, it is imprudent to directly compare the target ERPs as a function of spatial frequency in a masked priming design. However to get a feel for any overall differences between the spatial frequency conditions, we ran a supplementary set of analyses using a strategy that avoids the physical differences problem. Here we used difference waves computed by subtracting the unrelated and repeated conditions from each other. The logic here is that the subtraction within a condition removes any purely physical effect of a specific spatial frequency allowing for un-confounded comparisons of repetition effects across the three spatial frequency manipulations. What is lost in this analysis are main effects of repetition. ANOVAs were performed on these difference waves, in the same time windows specified above, with the three-level factor of Spatial Frequency (full, high, or low), Lexical Frequency (high or low) and the same distributional factors as used for the initial ANOVAs. The Geisser-Greenhouse correction was applied to all effects with more than one degree of freedom in the numerator (Geisser and Greenhouse, 1959).

## Results

### Full Spatial Frequency Results

**Figure 3:**
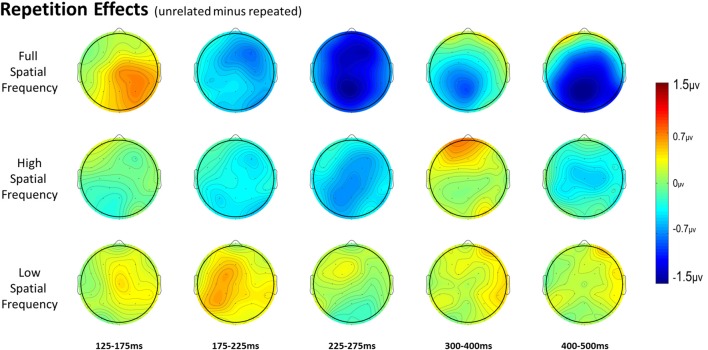
Voltage maps of the repetition effect (unrelated minus repeated) for each spatial frequency condition for all time windows.

**Figure 4:**
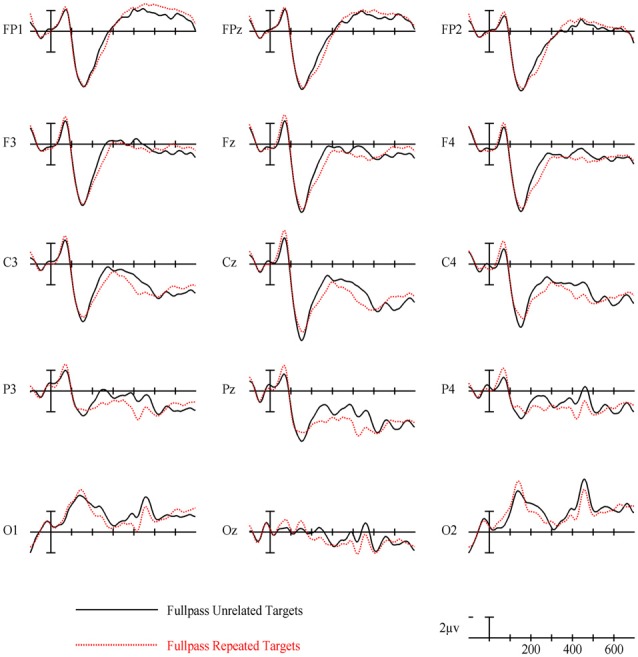
Event-related potentials (ERPs) for repeated vs. unrelated full spatial frequency primes.

**Figure 5:**
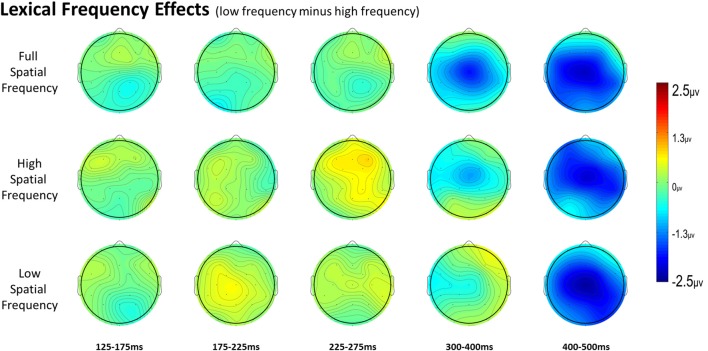
Voltage maps of the lexical frequency effect (low minus high) for each spatial frequency condition and all time windows.

For full spatial frequency primes during the earliest 125–175 ms epoch, there was no effect of repetition (*p* = 0.15). However starting in the 175–225 ms epoch there was a main effect of Repetition (*F*_(1,29)_ = 4.61, *p* = 0.04) which continued into the next 225–275 ms epoch (*F*_(1,29)_ = 25.51, *p* < 0.001) in the typical direction of unrelated words eliciting more negativity than repeated words (see Figure 3 for voltage maps and Figure 4 for ERPs). In the next epoch from 300 ms to 400 ms, the main effect of repetition fades (*p* = 0.19), though there was a marginal Repetition by Laterality interaction (*p* = 0.063) with repeated words still generating less negativity than unrelated words on the left side of the montage (see Figure 3). Also in this epoch began the main effect of lexical frequency (*F*_(1,29)_ = 4.28, *p* = 0.048), a Lexical Frequency by Anteriority interaction (*F*_(4,116)_ = 8.9, *p* < 0.001), and a Lexical Frequency by Anteriority by Laterality interaction (*F*_(8,232)_ = 3.29, *p* = 0.012) with low frequency words eliciting larger negativities than high frequency words in the center of the scalp (see Figure 5). In the final 400–500 ms epoch the Lexical Frequency main effect continued (*F*_(1,29)_ = 10.57, *p* = 0.003) along with the Lexical Frequency by Anteriority interaction (*F*_(4,116)_ = 5.6, *p* = 0.007) with a similar distribution to the previous epoch. Also in this epoch there was a main effect of Repetition (*F*_(1,29)_ = 5.71, *p* = 0.024) with unrelated words eliciting more negativity than repeated words (see Figure 3).

### High Spatial Frequency Results

**Figure 6:**
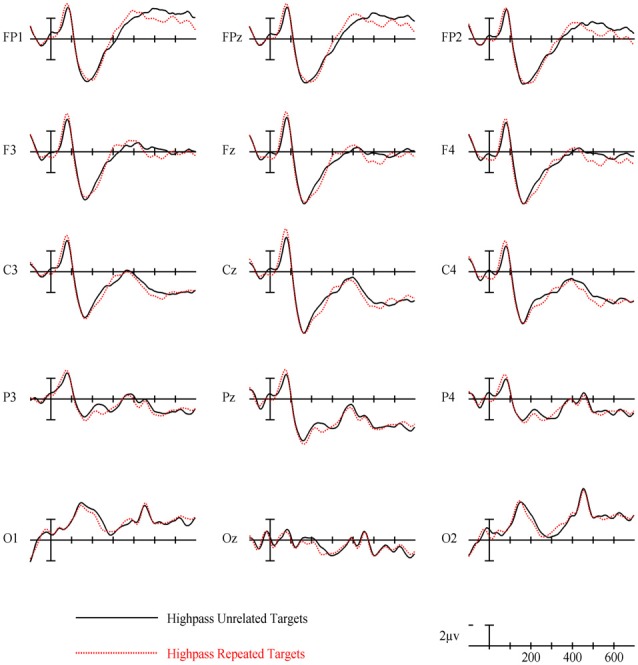
ERPs for repeated vs. unrelated high spatial frequency (HSF) primes.

**Figure 7:**
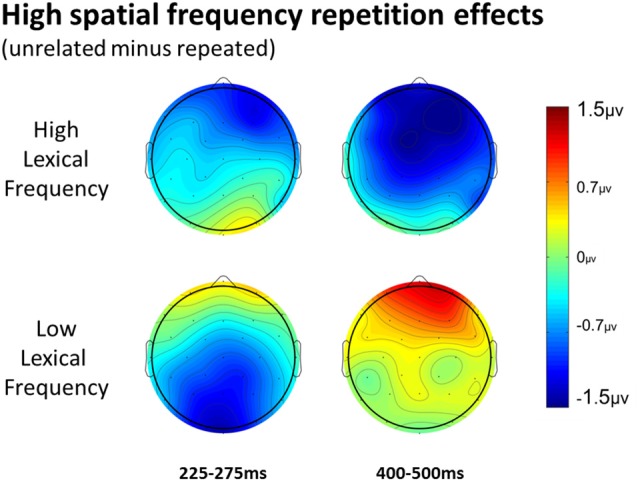
Voltage maps of the repetition effect for the 225–275 epoch and the 400–500 ms epoch for HSF primes, split between high and low lexical frequency pairs.

In the HSF prime conditions, there was no main effect of Repetition in the 125–175 ms epoch (*p* = 0.80), however there was a three-way Repetition by Laterality by Anteriority interaction (*F*_(8,232)_ = 2.97, *p* = 0.022) which indicates the presence of a small but consistent repetition effect in left anterior sites (see Figures 3, 6). In the next epoch 175–225 ms there was no main effect for repetition (*p* = 0.22). In the following 225–275 ms epoch there was still no main effect of Repetition (*p* = 0.12), however there was a significant three-way Repetition by Lexical Frequency by Anteriority interaction (*F*_(4,116)_ = 7.1, *p* = 0.004). Subsequent ANOVAs run separately for high and low lexical frequencies revealed that among high frequency words there was a three-way Repetition by Laterality by Anteriority interaction (*F*_(8,232)_ = 2.39, *p* = 0.041) driven by the repetition effect in right anterior sites (see Figure 7). Meanwhile for the low lexical frequency words in the same epoch, there was a two way Repetition by Anteriority interaction (*F*_(4,116)_ = 4.33, *p* = 0.023) likely due to a repetition effect at posterior sites (see Figure 7). In the following epoch, 300–400 ms, there was no main effect of Repetition (*p* = 0.22) but there was a three-way Repetition by Anteriority by Laterality interaction (*F*_(8,232)_ = 2.37, *p* = 0.042), seemingly due to a reversal of the previous repetition effects, with repeated words generating more negativity in left-anterior sites (see Figure 3). In the final 400–500 ms epoch there was again no main effect of Repetition, however there was a Repetition by Lexical Frequency interaction (*F*_(1,29)_ = 5.43, *p* = 0.027). Follow up ANOVAs revealed that for high lexical frequency items there was a marginal main effect of Repetition (*F*_(1,29)_ = 3.96, *p* = 0.056) in the direction of typical repetition effects, while there was no such effect for low lexical frequency items (*p* = 0.31), indicating that the Lexical Frequency by Repetition interaction was driven by a repetition effect for high lexical frequency words. Also in the 400–500 ms epoch there was a main effect of lexical frequency (*F*_(1,29)_ = 16.15, *p* < 0.001) and a three-way Lexical frequency by Laterality by Anteriority interaction (*F*_(8,232)_ = 3.39, *p* = 0.02) with low frequency items generating larger negativities than high frequency items especially in the center of the montage (see Figure 5).

### Low Spatial Frequency Results

**Figure 8:**
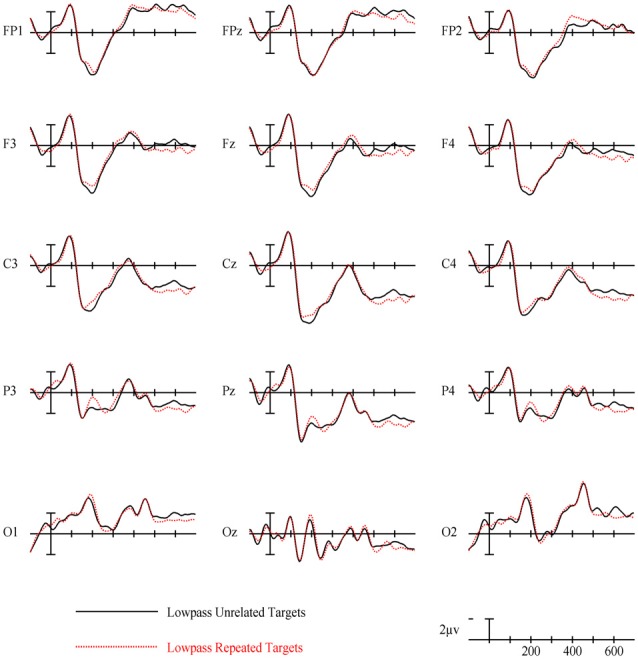
ERPs for repeated vs. unrelated low spatial frequency (LSF) primes.

In the earliest epoch, 125–175 ms, there was no main effect of Repetition (*p* = 0.34) for LSF primes. In the subsequent 175–225 ms epoch however, there was a significant main effect of Repetition (*F*_(1,29)_ = 5.05, *p* = 0.032). Interestingly, this effect was in the opposite direction of typical repetition effects, with repeated words generating more negativity than unrepeated words (see Figures 3, 8). For the remaining three epochs there were no other main effects of Repetition (225–275 ms: *p* = 0.85, 300–400 ms: *p* = 0.35, 400–500 ms: *p* = 0.49), or any interactions with Repetition. There was a Lexical Frequency by Laterality interaction in the 300–400 ms epoch (*F*_(2,58)_ = 5.11, *p* = 0.015) with low lexical frequency words generating larger negativities primarily on the left side of the montage. In the last 400–500 ms there was a main effect of Lexical Frequency (*F*_(1,29)_ = 17.67, *p* < 0.001) as well as a Lexical Frequency by Anteriority interaction (*F*_(4,116)_ = 4.03, *p* = 0.024) and a three way Lexical Frequency by Anteriority by Laterality interaction (*F*_(8,232)_ = 4.85, *p* < 0.001) indicating the typical centralized distribution of lexical frequency effects (see Figure 5).

### Difference Wave Results

In the 125–175 ms epoch there was only an interaction between Laterality and Anteriority (*F*_(8,232)_ = 3.31, *p* = 0.008), indicating a localized overall effect of Repetition with repeated words generating larger negativities than unrelated words in right posterior sites. In the next epoch, 175–225 ms, there was a main effect of Spatial Frequency (*F*_(2,58)_ = 5.83, *p* = 0.006), due to the beginning of the N250 repetition effect for the FSF and HSF conditions and the reversed effect for the LSF condition (see Figure 3 and above analyses). In the 225–275 ms epoch there was again a main effect of Spatial Frequency (*F*_(2,58)_ = 4.83, *p* = 0.014) from a strong N250 effect for FSF primes, a moderate effect for HSF primes, and no effect for LSF primes. Also there was a three-way Spatial Frequency by Lexical Frequency by Anteriority interaction (*F*_(8,232)_ = 4.07, *p* = 0.009) likely due to large difference in the distribution of the N250 for HSF primes as a function of Lexical Frequency (see Figure 7 and HSF results). In the 300–400 ms epoch, there was only a marginal effect of Anteriority (*F*_(4,116)_ = 3.17, *p* = 0.059) and a marginal interaction between Laterality and Anteriority (*F*_(8,232)_ = 2.17, *p* = 0.061), likely due to a combination of the beginning of the N400 repetition effect for FSF primes and the anterior reversed effect for HSF primes. In the last 400–500 ms epoch, there was an effect of Anteriority, showing the overall N400 repetition effect in central-posterior sites (see Figure 3). There was also an effect of Lexical Frequency, reflecting that the N400 repetition effect was larger for high lexical frequency pairs than low lexical frequency ones. Additionally there was a marginal effect of Spatial Frequency (*F*_(2,58)_ = 2.62, *p* = 0.097), and a marginal interaction between Spatial Frequency and Anteriority (*F*_(8,232)_ = 2.53, *p* = 0.065), reflecting the large N400 effect for FSF primes, a smaller N400 effect for HSF primes and no effect for LSF primes (see Figure 3).

### Behavioral Results

On average, participants correctly identified animal words in the target position in 89% of trials (FSF primes: 86%, HSF primes: 91%, LSF primes: 90%) and animal words in the probe position in 2% of trials. On average participants incorrectly identified a target word as an animal on 3% of trials.

## Discussion

As predicted, FSF primes produced the typical pattern of masked priming effects, with an attenuated N250 and N400 to repeated words. Also as hypothesized, HSF primes produced effects on the N250 which interacted with lexical frequency, though the effect on the N400 was only marginal and carried by high lexical frequency words. Interestingly there was also a reversal of the typical direction of the repetition effect at about 300 ms for HSF primes. LSF primes on the other hand did not show any indication of normal priming effects in the N250 or N400 windows, although they did produce one distinct effect; a reversed effect of repetition at about 200 ms, exhibiting greater negativity to repeated words than unrelated words.

### Full Spatial Frequency

Overall, the findings for FSF primes followed from prior research using ERPs to investigate masked priming (e.g., Holcomb and Grainger, 2006; Kiyonaga et al., 2007). Similar to the N250 effect found by Holcomb and Grainger (2006), there was a clear effect of repetition in the 175–225 ms and 225–275 ms epochs with repeated words producing less negativity than unrelated words and this effect was widely distributed across the midline of the montage (see Figure 3). Also parallel to previous studies there was a pronounced reduction in N400 amplitude to repeated words. This pattern of attenuated N250 and N400 activity is generally thought to represent the reduced amount of processing necessary to activate a word’s lexical-semantic networks if it has been primed (Holcomb and Grainger, 2006). Contrary to Holcomb and Grainger (2006) however, we did not find N/P150 effects for FSF primes. The N/P150, thought to represent early visual feature mapping process, is more positive over right occipital sites and more negative over frontal sites to repeated words (Holcomb and Grainger, 2006; Chauncey et al., 2008; Dufau et al., 2008). Given the dependence on feature overlap for the elicitation of the N/P150 (Grainger and Holcomb, 2009), it is likely that it was not found in the current study because of the limited featural overlap in the repeated condition due to the case change, and perhaps also that there were fewer prime-target pairs than in other N/P150 studies. Overall, the pattern of effects after the N/P150 epoch for FSF primes replicates previous studies, again indicating the presence of N250 and N400 repetition effects. Since the same words were used for the other spatial frequency conditions, these findings guide our interpretation of the HSF and LSF results.

### High Spatial Frequency

HSF primes elicited some similar effects to those seen in typical ERP masked priming studies, though there were some different patterns of effects as well. First, there was an interaction between repetition and electrode site in the first epoch of 125–175 ms with repeated words eliciting less negativity than unrelated words in left posterior sites (see Figure 3). This pattern is interesting because it is similar in its timing to an N/P150 effect. However, it is likely not due to the same neural activity that produces typical FSF N/P150 effects (e.g., Holcomb and Grainger, 2006) since it is the opposite polarity and has a different spatial distribution. Nevertheless, this is the epoch in which early visual feature processing is thought to occur (Grainger and Holcomb, 2009) so one tentative hypothesis is that this effect reflects a unique contribution of HSF information to early featural processing. Consistent with this possibility is the evidence that left hemisphere visual areas respond preferentially to HSF information (e.g., Woodhead et al., 2011; Ossowski and Behrmann, 2015).

More comparable to typical masked priming effects, there was evidence of an N250 repetition effect with HSF primes (see Figure 6), which was similarly distributed as the similar effect reported above for FSF primes, though smaller (see Figure 3). Given the similarity of the two N250s, this provides evidence that HSF information is being used by at least part of the same sublexical processing system utilized by normal word recognition. Interestingly, and unlike in the FSF condition, this N250 repetition effect between 225–275 ms interacted with lexical frequency and anteriority indicating a larger repetition effect in anterior sites for high lexical frequency word pairs, and a larger effect in posterior sites for low lexical frequency word pairs (see Figure 7). These different foci of the effect could point to distinct processes which make up the normal N250 effect. The more typical N250 effect has a strong anterior component which may be driven by later processes relating to the interface with whole word orthographic representations thus affected by priming manipulations like full repetition (e.g., the FSF repetition effect in the current study; Holcomb and Grainger, 2006; Kiyonaga et al., 2007), or orthographic neighbors (Massol et al., 2011, 2012). However Grainger et al. (2006) found that transposed letter primes (e.g., brain-barin) had an earlier, more posterior effect on the N250 while pseudohomophone primes (e.g., brain-brane) had the typical later, more anterior effect. They hypothesized that the transposed letters influenced earlier sublexical processing (perhaps at the level of bigrams or trigrams) while the pseudohomophone primes began to affect processing later, when the target’s orthographic code is translated into phonological code (for more on bimodal interaction, see Grainger and Ferrand, 1994). In the current study, the anterior focused high lexical frequency repetition effect may represent influence at higher level, perhaps while sublexical orthographic processing output is being mapped onto full word representations, indicating that the HSF primes were able to activate some sort of higher level representation. Meanwhile the low lexical frequency primes were slower to, or not able to activate higher representations, stalling information flow in lower level networks. Thus the HSF repeated primes for the low lexical frequency pairs more so affected processing of the target in lower sublexical processes, while the repeated HSF primes for high lexical frequency pairs were able to aid in further-along computations during this epoch. As indicated by subsequent effects discussed below, whatever processing repeated HSF primes aided for the high lexical frequency pairs seems to have been more advantageous to the eventual recognition of the target. This interaction was not found in the current FSF prime condition since the more visible primes might be able to more fully activate the entire network regardless of lexical frequency, resulting in no significant interaction (*p* = 0.36).

Following the N250 effect was an effect during the 300–400 ms epoch in the opposite direction with greater positivity to repeated words, which was focused on left anterior sites (see Figure 6). At least two components sensitive to similar manipulations have been identified in this time frame. Chauncey et al. (2011) report a P350 effect related to lexical frequency switching, elicited by low lexical frequency targets preceded by unrelated high lexical frequency primes. Holcomb and Grainger (2006) report a P325 effect only to fully repeated words, which has been interpreted as representing processing within whole-word representations. Since it is in the opposite direction (more negativity to repeated words), the effect currently observed does not fit well with either of these previously seen components. One possibility is that this reversal relates to the reduced, but still observable, effects of HSF primes. As with the FSF primes, repeated words should be processed more efficiently, though perhaps this advantage would be smaller for HSF primes than FSF primes. Conversely, with the lessened impact of the HSF primes, the unrelated primes would interfere less by not as strongly activating irrelevant words. Thus, due to less interference of unrelated HSF primes, but still some processing advantage of repeated primes, we see a reversal which intercedes the N250 and the N400. Evidence of this can be seen with the FSF primes (and other masked repetition priming ERPs) as a reduction of the repetition effect in this epoch. However the more salient primes better activate both repeated and unrelated information, leading to larger overall negativities to unrelated information spanning across the N250 and N400, which might obscure smaller or shorter components related to repeated words.

Finally, lexical frequency effects began for the HSF condition in the 400–500 ms epoch, later than in the FSF condition, likely because of less influence by the primes, which were also high or low lexical frequency according to the frequency condition of the target. An interaction between repetition and lexical frequency was also found in this epoch such that high lexical frequency prime-target pairs showed evidence of an N400-like repetition effect but low lexical frequency pairs did not. This pattern was similar to what was observed in the FSF condition although for HSF, there was no indication of an N400 repetition effect for low lexical frequency words (see Figure 7) while with FSF primes, low frequency word pairs also showed the effect. These results suggest that HSF information in a 50 ms prime is enough to activate a word to the point of gaining a processing advantage during the N400 of a repeated word. However, the signal from the HSF primes is weaker, perhaps because a substantial portion of the luminance information has been filtered out, and thus can only activate high lexical frequency words. Further, this discrepancy in N400 effects supports the explanation that the earlier lexical frequency interaction may be due to HSF information reaching relevant word recognition systems, but not as strongly and thus only activating higher lexical frequency words.

### Low Spatial Frequency

LSF primes did not produce any of the ERP effects typically found with masked repetition priming. This is perhaps not surprising since even long duration LSF words are difficult to read and therefore less likely to activate their lexico-semantic representations sufficiently to modulate a component like the N400. Effects on earlier components may be more plausible since these reflect lower-level feature/lexical processing which might have received enough activation from weak LSF information. However, there was not a typical N250. Rather, in an early N250 window (175–225 ms) LSF unrepeated words actually produced a larger positivity than repeated words. This effect, which is the opposite polarity of the traditional masked priming N250 effect but is the same polarity as the positive phase of the earlier N/P150, was concentrated over left-posterior sites around electrode site P3 (see Figure 8). This is different than both the typical N250 and N/P150 distributions leaving it unclear whether this effect has more to do with the physical properties of the prime-target pair (like the N/P150) or more abstracted sublexical or lexical properties of a prime-target pair (like the N250). The direction of the effect matches the posterior aspect of the N/P150, although given the case change between the repeated primes and targets, featural overlap was only partial. Nonetheless, it is still possible that this effect reflects some sort of bottom-up response to repeated LSF features. Alternatively, the timing of the effect matches better with a sublexical-lexical component such as the N250, perhaps earlier than the typical N250 because it is operating on LSF information. Following the role of LSFs in Bar’s model of object recognition, if this component is related to lexical-level processing, it might represent the matching of a fed-back LSF lexical representation or prediction to the incoming repeated FSF target information. Of course more research is necessary to have anything more than speculative ideas about what this early repetition effect with LSF primes reflects.

The main effect of lexical frequency began in the 400–500 ms epoch for the LSF prime condition, later than the FSF condition. Similar to why the effect was later for HSFs than FSFs, LSF primes were probably less capable of activating lexical information, hence the lexical frequency of the LSF primes did not affect the processing of target words as much as the FSF primes. There was no significant effect of repetition in any of the later epochs for LSF primes, suggesting minimal influence of LSF primes on the N400. This can be taken as evidence that under these conditions (i.e., foveal presentation, 5 letters, a prime-target case change and a 50 ms duration), LSF primes do not affect word recognition processes up to the level of semantic access.

### Difference Waves

In the 125–175 ms epoch, we see an effect of Repetition across all spatial frequencies and focused on right-posterior sites. This effect is likely carried by the FSF condition which was almost significant by itself, but was helped by a similarly patterning effect for the LSF primes. Interestingly in this epoch, the LSF primes produced more similar effects to the FSF primes than the HSF primes, which produced their own pattern (discussed above). This may indicate that previously observed form-based early components like the N/P150 are not elicited with HSF primes, and instead rely on the contributions of medium or lower spatial frequencies. In the following 175–225 ms epoch there was an interaction between the Repetition effect and Spatial Frequency, due to the more typical early N250 effects of the FSF and HSF primes, and the significant reversal of the effect for LSF primes (see Figure 3). This strongly suggests that repeated LSF and HSF information are being treated in different ways at this level of processing. While the HSF effect mirrors that of the FSF condition, the LSF primes seem to be producing a previously unobserved component reflecting an unknown process (discussed above).

In the later 225–275 epoch there was again an interaction between Spatial Frequency and Repetition, and an interaction with Lexical Frequency as well. Again, the FSF and HSF conditions seem to pattern similarly here, while the LSF condition now shows no evidence of any effect in this epoch. Further, as discussed above, the distribution of the HSF N250 is dependent on the lexical frequency of the prime-target pair (see Figure 7), while the same is not true for the FSF pairs, perhaps indicating a limited ability for HSF information to activate all lexical representations. In the 300–400 ms epoch, the difference wave analysis only revealed marginal overall interactions between Repetition and distributional variables, likely due to the localized beginning of the N400 effect for FSF primes and the anterior reversal of the effect for HSF primes (discussed above). In the final 400–500 ms epoch, there was an overall effect of Repetition due to the N400 effect present in both the FSF and the HSF conditions. This effect had a marginal interaction with Spatial Frequency, likely due to the lack of an N400 effect for LSF primes and only a small N400 effect for HSF primes which was only present for high lexical frequency words. Overall this suggests that N400 priming requires more than just the lowest or highest spatial frequencies, though if the word is advantaged by being frequently used, it may be activated by only HSF information.

### Conclusions

Words fundamentally require more precise and complex processing than most other categories of visual stimuli. Perhaps then it is conceivable that word recognition might defy the normal pattern of coarse-to-fine processing that most other visual stimuli are thought to employ (e.g., Schyns and Oliva, 1994; Bar et al., 2006; Goffaux et al., 2011). Overall, the current findings suggest that indeed, HSFs are more salient for the neural mechanisms that underlie fast visual word processing as indexed by masked priming ERP effects. This interpretation is consistent with the hypothesis that visual word recognition operates mainly on higher spatial frequencies. That said, the repetition effects with HSF primes were markedly weaker than with FSF primes, indicating that useful spatial frequencies were missing. Further, the repetition effects were largely carried by the high lexical frequency pairs, again demonstrating that frequently used words are somehow privileged during word recognition. These interactions may also inform the functional significance of the N250 effect, specifically aspects of its timing and widespread distribution, as being the product of multiple functions potentially including sub-lexical featural processes, the compilation of a specific ordinal orthographic code, and the mapping of this code onto higher level lexical representations. In the framework of the bi-modal interactive activation model, these findings indicate that the spatial frequency content of a prime affects the degree to which it can activate lexical information. This access is further modified by the lexical frequency of the prime, perhaps due to higher resting state activity of higher frequency items, incurring a processing advantage as early as the sub-lexical level, indicating feedback mechanisms. Similarly, in a predictive coding framework, these findings indicate that the spatial frequency as well as the lexical frequency of a prime impact the ability for higher level networks to predict incoming information, with the least amount of prediction error to high lexical and spatial frequency primes, compared to low spatial or low lexical frequency primes.

LSFs did not produce any evidence of typical ERP masked repetition priming effects such as the N400 or N250, suggesting minimal influence on the typical word recognition process. They did however elicit a distinct effect which indicates that LSF information affected the processing of the target word in some way. The effect around 200 ms is difficult to interpret, but could represent a (partial) featural overlap effect in some LSF information processing stream, or an interaction between bottom-up target processing and fed-back word-shape information or predictions about the prime. Overall it does not seem to be the case that LSF primes benefit or interfere with the processing of target stimuli during the normal course of word recognition. However the presence of the early effect does indicate that LSF information is being processed by word recognition systems at some level.

Besides the full spatial frequency condition, the current study only had one condition with the highest spatial frequencies and one with the lowest, neither of which accounted for the full pattern of masked repetition priming effects. Thus, further research using more specific bands of spatial frequency information is necessary to determine the exact range of frequencies used by word recognition systems. Further, we used a case change between prime and target pairs, which emphasizes priming at a more abstracted lexical level, but leaves open questions about form-level priming. Additionally, this study only used five letter words presented centrally, which likely advantaged the HSF condition compared to the LSF condition. Longer words or words presented in the periphery may benefit more from LSFs, while HSF processing likely becomes more inefficient as word length and distance from fixation increases. Thus, future research may reveal greater contributions of lower spatial frequencies with other paradigms which better mimic the actual process of reading. This could also better explain the connection between spatial frequency and dyslexia, which is indeed an impairment of the entire reading process, not just word recognition.

In sum, HSF primes demonstrated a greater ability than LSF primes to elicit the typical series of masked repetition priming ERP effects. Assuming this pattern of effects represent steps in the word recognition process, this finding adds further evidence that HSFs, and therefore smaller features contribute more so to word recognition than global information, at least at the level of single words. However, LSFs were also seen to produce a distinct effect which may indicate that larger global information still informs the process of word recognition.

Visual systems have evolved for millions of years to reach the current level of efficiency which seems to be reliant on multiple interacting pathways operating on both rough LSF and precise HSF information. If, as accumulating evidence suggests, reading really does depend on mostly HSF information, then this speaks to the incredible adaptability of the human brain to create new architecture for a behavior that has only existed for a few 1000 years. Nevertheless, it is perhaps still too hasty to dismiss the possible role of larger scale LSF information during word recognition.

## Author Contributions

PJH, JG and KJM designed the study. KW collected and analyzed the data. KW, PJH and JG participated in the writing and revising of the manuscript.

## Conflict of Interest Statement

The authors declare that the research was conducted in the absence of any commercial or financial relationships that could be construed as a potential conflict of interest.
